# web-rMKL: a web server for dimensionality reduction and sample clustering of multi-view data based on unsupervised multiple kernel learning

**DOI:** 10.1093/nar/gkz422

**Published:** 2019-05-22

**Authors:** Benedict Röder, Nicolas Kersten, Marius Herr, Nora K Speicher, Nico Pfeifer

**Affiliations:** 1 Methods in Medical Informatics, Department of Computer Science, University of Tübingen, 72076 Tübingen, Germany; 2 FZI Forschungszentrum Informatik, Microelectronic System Design (SiM), 76131 Karlsruhe, Germany; 3 Institute for Translational Bioinformatics, University Hospital Tübingen, Germany; 4 Department of Computational Biology and Applied Algorithmics, Max Planck Institute for Informatics, Saarland Informatics Campus, 66123 Saarbrücken, Germany

## Abstract

More and more affordable high-throughput techniques for measuring molecular features of biomedical samples have led to a huge increase in availability and size of different types of multi-omic datasets, containing, for example, genetic or histone modification data. Due to the multi-view characteristic of the data, established approaches for exploratory analysis are not directly applicable. Here we present web-rMKL, a web server that provides an integrative dimensionality reduction with subsequent clustering of samples based on data from multiple inputs. The underlying machine learning method rMKL-LPP performed best for clinical enrichment in a recent benchmark of state-of-the-art multi-view clustering algorithms. The method was introduced for a multi-omic cancer subtype discovery setting, however, it is not limited to this application scenario as exemplified by a presented use case for stem cell differentiation. web-rMKL offers an intuitive interface for uploading data and setting the parameters. rMKL-LPP runs on the back end and the user may receive notifications once the results are available. We also introduce a preprocessing tool for generating kernel matrices from tables containing numerical feature values. This program can be used to generate admissible input if no precomputed kernel matrices are available. The web server is freely available at web-rMKL.org.

## INTRODUCTION

With advances in sequencing and computer technology, we are now able to extract multi-omic data and analyze them efficiently. Multi-omic data consist of several measurements of different biological layers, e.g., the genome, proteome, transcriptome, epigenome or microbiome. Combining them can reveal deeper insights about the state of an organism, which could be used in personal medicine to find the most suitable therapy. In recent years, several algorithms have been developed to process these data and extract meaningful information. In cancer treatment, a common task is to group patients into homogeneous clusters, where patients within a cluster might have similar survival times or similar responses to a certain therapy. Thus, results of these algorithms could be helpful in treatment decision making in order to find the most promising treatment for each patient.

Multiple kernel learning is often used in the context of multi-omic data analysis because of its ability to integrate data of various types (e.g. numerical and sequence data) and characteristics. rMKL-LPP is a representative of unsupervised multiple kernel learning (1). The technique enables the identification of sample clusters by learning an integrative dimensionality reduction. In 2018, Rappoport and Shamir published the first benchmark for multi-omic and multi-view clustering, comparing state-of-the-art algorithms in the number of enriched clinical labels and significance in survival times between clusters (2). For evaluation, nine algorithms were applied to ten different tumor multi-omic datasets. Each dataset contained three types of omics data: gene expression, DNA methylation and miRNA expression. The number of patients within a dataset ranged from 170 to 621. Although no algorithm consistently outperformed all others, rMKL-LPP performed best with respect to the overall number of significant clinical parameters and was among the top-3 with respect to survival (1. MCCA, 2. MultiNMF) (3,4). These algorithms are provided as standalone programs or packages, requiring prior setup and sufficient computing power. This reduces their practical use whereas an intuitive web-application could make them more accessible.

To the best of our knowledge, none of the benchmarked methods offers a web server. The only existing web services in that field are mixOmics (mixomics.org) and BioNMF (5). BioNMF applies a non-negative matrix factorization on a single input file (5). However, it does not make use of multiple omic files. This could be a crucial shortcoming as multi-omic data may provide substantial information, which can result in a more precise clustering. Therefore, MultiNMF (4) was developed, for which no web server is available. MixOmics on the other hand offers several methods like PCA, rCCA or PLS for dimensionality reduction of multi-omic data. Although they still provide their R package via Bioconductor, their web-interface was not accessible anymore at the time of writing this manuscript.

In this work, we present web-rMKL, a web server that employs a state-of-the-art algorithm for processing and clustering of multi-omic data, which is currently the only in its field. Web-rMKL emphasizes an intuitive handling of uploading data and individual parameter setting. Default parameters are provided based on the evaluation of Speicher and Pfeifer (1). Users can choose to receive notifications by e-mail once the results are available. The web server is completely free and no log-in is required. However, for more convenience users can register to obtain an overview of previous submissions and directly download previously computed results.

## MATERIALS AND METHODS

### Format of input files

rMKL-LPP requires at least two input files. One is a text file containing identification strings for each sample, whereas the other is a MAT file (binary MATLAB^®^ file format) of a precomputed kernel matrix. Since the algorithm works on single-omic as well as multi-omic data, several MAT files can be supplied, where each file contains a kernel matrix for one specific data type (i.e. set of features) or kernel function. Furthermore, admissible input matrices have to be positive semi-definite and symmetric. This precondition is also checked on the server after uploading all input files. Matrices that are not positive semi-definite will be transformed to fulfill this condition by subtraction of the smallest Eigenvalue from the diagonal, as shown by Vert *et al.* (6).

For the case that no MAT files are available we developed an offline preprocessing tool. This tool can process CSV files and outputs admissible MAT files. The input for this tool can either be a table with numerical feature values or an already precomputed kernel matrix in a CSV table. In the latter case, the tool checks if the kernel matrices are symmetric and converts them into MAT files. If a CSV file with raw numerical feature values is provided, the user can compute kernel matrices based on customizable parameters and export them as MAT files. In both cases, the offline tool also extracts and exports the sample identifiers, if they are available in the CSV files, such that the generated identifier file can be directly used as input for web-rMKL.

### Workflow

**Figure 1.**
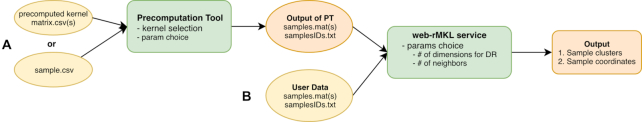
Workflow describing input and output of web-rMKL services. Yellow boxes represent input files provided by the user; orange is output generated by our services and in green our provided services. Two starting points are possible: (**A**) Offline: Several precomputed kernel matrices (CSV format) or feature files are input of the precomputation tool. Currently, the computation of RBF, polynomial and linear kernel are supported. The kernel parameters *Gamma, Coefficient* and *Degree* (depending on kernel choice) can be set. Output of the precomputation is a valid input for the online service. (**B**) Online: Direct upload of sample identifiers and kernel matrices (MAT format) is possible to use the rMKL-LPP service. The results are generated based on the choice of parameters and can be visualized online (for two and three dimensions). General output is two text files: 1) the learned cluster assignment for each sample, and 2) the coordinates of the sample projections.

The pipeline can be split into an offline preprocessing and an online rMKL-LPP part. An illustration of this process can be seen in Figure 1. The first step is the offline generation or conversion of the input files, which can be skipped if precomputed kernel matrices stored as MAT files are already available. In the main part, the set of kernel matrices is processed in web-rMKL, resulting in dimensionality reduction and clustering of the multi-omic data.

#### Web-rMKL

Once the user uploaded the sample ID file and admissible kernel matrices, two computation parameters have to be selected. The number of neighbors $k_\mathcal {N}$ and the number of dimensions *n* that the samples should be projected onto. The default values are nine nearest neighbors and three projection dimensions. Optionally, the user can supply an e-mail address to receive a notification once the results are available. Afterwards, the job will be queued and processed when enough resources are available. At this point, further jobs can be submitted. Web-rMKL then applies the rMKL-LPP algorithm to the supplied data. The algorithm performs a robust joint dimensionality reduction that aims to conserve the distances of each sample to its $k_\mathcal {N}$ nearest neighbors. The output consists of two text files. The first file assigns a cluster ID to every sample ID. The second file lists the coordinates for each sample ID in the *n*-dimensional output space. Registered users can also see a history of their previous submissions to access them more easily.

#### rMKL-LPP

The joint dimensionality reduction technique rMKL-LPP was introduced in 2015 by Speicher and Pfeifer (1). The method builds on multiple kernel learning for dimensionality reduction (MKL-DR), which was introduced by Lin *et al.* (7), making it more robust by adding a regularization term. MKL-DR itself is based on the graph embedding framework, introduced by Yan *et al.* (8). This framework facilitates the implementation of a large variety of different dimensionality reduction techniques. By using the kernelized version, dimensionality reduction can be performed in a non-linear manner. The combination of graph embedding with multiple kernel learning enables applying these dimensionality reduction schemes to multi-omic data sets. In this setting, data integration is achieved by learning a weighted linear combination of the kernel matrices, which represent the information of the different data types. For the moment, our server is restricted to locality preserving projections (LPP) in combination with rMKL, since we and others have shown state-of-the-art performance with that combination (rMKL-LPP) for several datasets (1,2). Based on the learned projection of the samples in the *n*-dimensional space, *k*-means is used to identify the sample clusters. The number of clusters *k* is determined using the average silhouette score.

#### Preprocessing tool

The preprocessing tool provides the offline computation of kernel matrices using three different methods with custom parameter selection, as well as validation of precomputed kernel matrices in CSV format.

For two samples *x*_*i*_ and *x*_*j*_, the Gaussian radial basis function (RBF) kernel is calculated by

\begin{equation*} k_{\rm RBF}(x_{i}, x_{j}) = \rm exp(-\gamma\Vert x_{i}-x_{j}\Vert^{2}), \end{equation*}

where the parameter γ can be adjusted to values >0 by the user. As a default setting, the general rule of thumb

\begin{equation*} \gamma _{\text{default}} = \frac{1}{2p^2} \end{equation*}

will be applied based on the number *p* of features (9). For the computation of the polynomial kernel:

\begin{equation*} k_{\text{poly}}(x_i, x_j) = (\gamma \left\langle x_i,x_j\right\rangle + cost)^{d}, \end{equation*}

the kernel slope γ, the constant *cost* factor and the polynomial degree *d* can be chosen (default: γ = 1, *cost* = 0, *d* = 3). As a third option, the linear kernel can be selected, which corresponds to the dot product of the input vectors:

\begin{equation*} k_{\text{linear}}(x_i, x_j) = \left\langle x_i,x_j\right\rangle \end{equation*}

and does not require further input parameters.

As described above, the input CSV files must contain a set of numerical features as columns and the values for each sample as rows. Furthermore, the sample ID tag can be provided in the first column of the file. In addition to the MAT files containing the kernel matrices, the sample IDs will also be exported to a text file such that the output of the offline preprocessing tool is ready for use on the web-rMKL server.

Based on precomputed, unlabeled kernel matrices in CSV files, the preprocessing tool can validate and export MAT files for use on the server. The validation step checks for symmetry of the imported matrices.

## RESULTS

### First example use case—evaluation of head and neck squamous cell carcinoma survival times

In the initial publication of the rMKL-LPP algorithm by Speicher and Pfeifer (1), the application of this unsupervised learning method was cancer subtype discovery. In this first example, we demonstrate a possible output of the application of the complete web-rMKL workflow on a head and neck squamous cell carcinoma (HNSC) dataset (10).

Based on multi-omic data of four different types (gene expression, DNA methylation, miRNA expression and copy number variation, described in Supplementary File 1), a clustering of the 462 patients in the dataset was obtained. For each data type, one kernel matrix was computed with the offline precomputation tool using the RBF kernel with default parameters. The resulting files were then used as input for the web-rMKL server. The output of the online computation with nine nearest neighbors and five projection dimensions (which was the default parameter setting suggested in the original publication) yielded three clusters. A subsequent survival analysis based on the Kaplan–Meier estimation has shown differences in survival times for the identified patient clusters that were however not significant (*p* = 0.24 in log-rank test with Bonferroni correction for multiple testing). Due to differences in available follow-up times for different patients, the survival analysis was limited to the first five years after diagnosis (1825 days). To figure out whether a smaller subset of patients with significantly different survival times could be identified, the web-rMKL computation was repeated with different parameters. A refinement of the clustering was obtained by reducing the number of nearest neighbors to 5. As a result, four clusters of smaller sizes were discovered.

**Figure 2.**
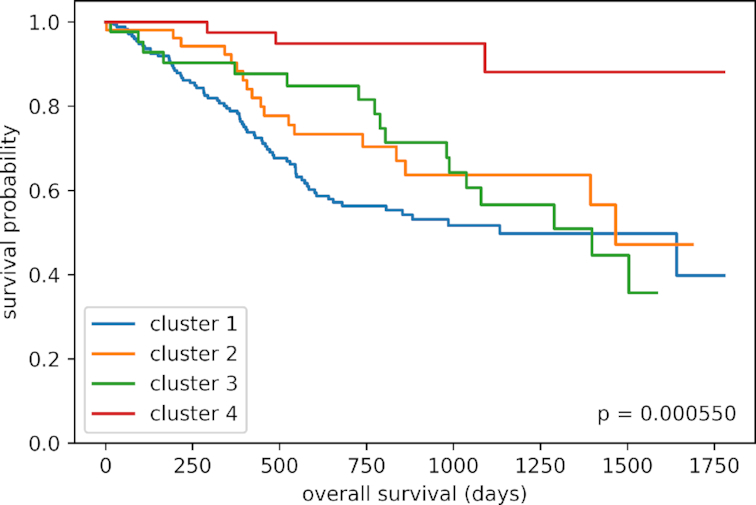
First example use case: Survival analysis of four head and neck squamous cell carcinoma patient clusters shows significant difference in 5-year survival (*p* = 0.0006, log-rank test with Bonferroni correction for multiple testing). Cluster 4 shows highest survival probability within the first 5 years. 64% of patients in cluster 4 received targeted molecular treatment while only 30–38% of individuals in cluster 1–3 received such a treatment.

As shown in Figure 2, the 5-year survival analysis for the clusters shows significantly different overall survival rates (*p* = 0.0006 in log-rank test with Bonferroni correction for multiple testing). Further exploration of the available clinical data revealed that the majority of patients within the cluster with the best overall survival rates within the first five years (cluster 4) received targeted molecular treatments (64%) while most patients within the other clusters did not receive this kind of medication. This exemplifies two things. First, our method seems to be able to group patients with similar characteristics that might be relevant for treatment together, because one of the simplest explanations for the lack of treatment for most of the patients from the other clusters is that there was no targeted therapy available. Second, our method might help to uncover underlying confounders while looking at the different data modalities jointly. For this use case, the application of the integrative dimensionality reduction of the four data types coupled to k-means was able to produce clusters with significantly different clinical outcome (survival rate).

### Second example use case - stem cells and derived differentiated lines

**Figure 3.**
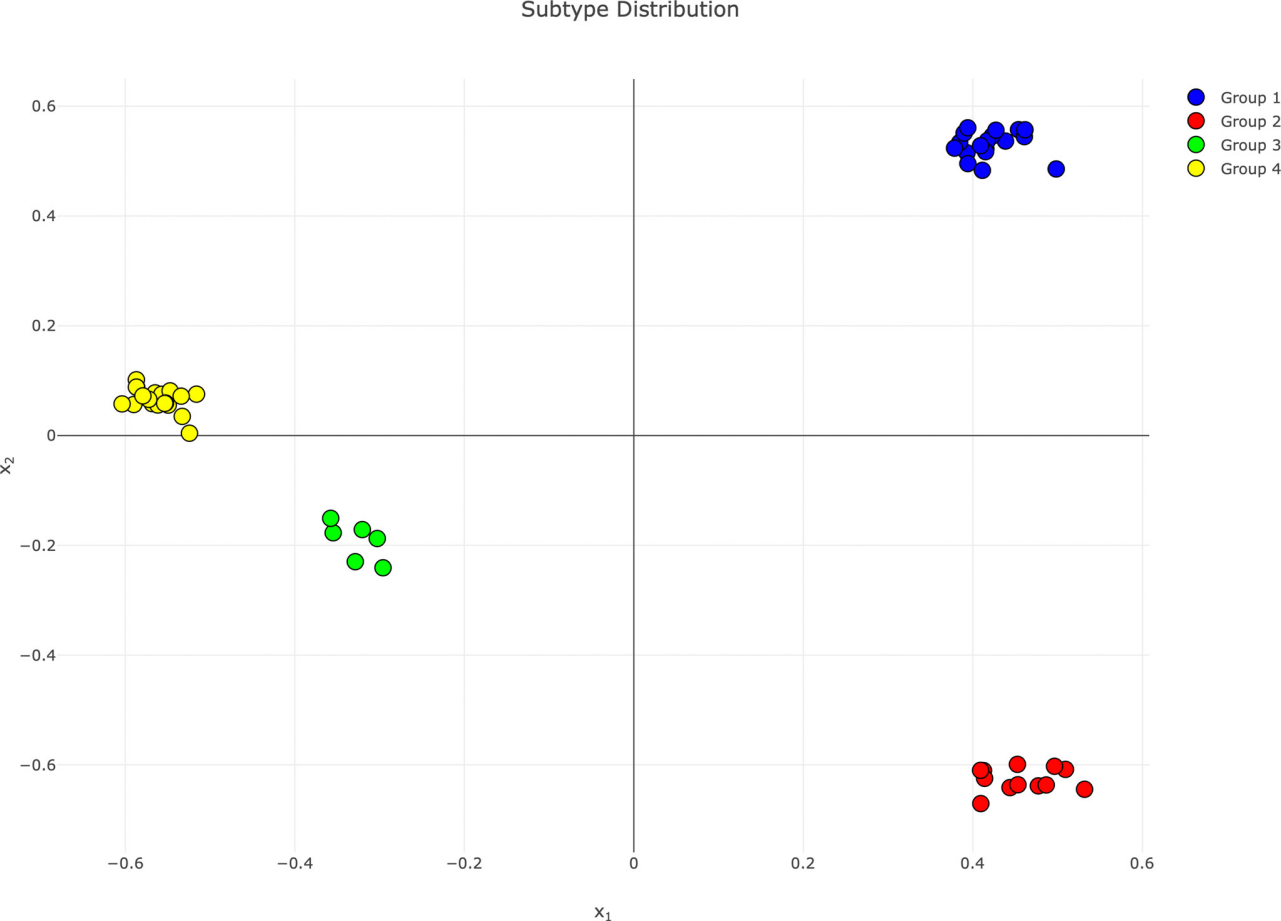
Second example use case: Stem cells and derived differentiated lines. The identified clusters correspond to differentiation states: definitive endoderm + mesoderm (Cluster 1), embryoid body (Cluster 2), ectoderm (Cluster 3) and stem cells (Cluster 4). This figure shows the unmodified output visualization of web-rMKL.

In this application scenario, we analyzed data characterizing human stem cells (embryonic and induced pluripotent stem cells) and derived differentiated lines (11). Induced pluripotent stem cells (iPSC) have attracted considerable attention due to their potential role in various fields including regenerative medicine and drug discovery (12). However, prior to experimental usage or transplantation of the iPSC or derived differentiated lines, analyses are necessary to validate their quality and, consequently, ensure reproducibility of subsequent experiments. For each sample, the given dataset provided measurements for three data types (gene expression, DNA methylation, and miRNA expression, described in Supplementary File 1). One kernel matrix per data type was generated by the offline precomputation tool using the RBF kernel with the default parameter setting. For the application of web-rMKL, the number of nearest neighbors was set to five to account for the small size of the dataset (57 samples). For visualization purposes, the number of dimensions was reduced to two. The integrative dimensionality reduction reveals a clear cluster structure in this dataset (see Figure 3). The resulting clustering reflects the differentiation states of the samples: Cluster 1 consists of 9 samples of definitive endoderm (DE) and 9 samples of mesoderm (MESO), Cluster 2 consists of the 12 samples of embryoid body, Cluster 3 consists of the six ectoderm samples, and Cluster 4 consists of the 21 stem cells. Except for DE and MESO, the integrative analysis enables a perfect separation according to the differentiation state without having used this type of information in the process.

## DISCUSSION

To the best of our knowledge, web-rMKL is the only available web server for integrative dimensionality reduction of multi-omic data. We provide a complete workflow starting with raw data and resulting in a set of sample clusters. Splitting the pipeline into an offline and online part significantly increases the data privacy. The CSV files that are used for kernel computation contain information for each sample or patient, which might be sensitive information. However, the kernel matrices that are uploaded to the server only include pairwise sample similarities and are thus insensitive. Therefore, the separation into offline kernel computation through our preprocessing tool and online dimensionality reduction followed by clustering provides the user with the performance and convenience of the server computation while the raw data is kept private on the computer of the user.

In the Results section, we demonstrated two possible applications of web-rMKL showing the capabilities of the integrative dimensionality reduction that is performed on this web server. While the cluster output was used for a subsequent analysis in the first use case, the results of the second use case were directly retrieved from the output of the server. As with any method, the application of the web-rMKL workflow also depends on the characteristics of the input dataset and thus, the input parameters might have to be modified accordingly, which is easily possible in the web-interface. This also applies to the selection of kernel models and kernel computation parameters.

## CONCLUSION

We have developed web-rMKL to offer an easy to use web-interface for a state-of-the-art dimensionality reduction algorithm with subsequent clustering of multi-omic data. To the best of our knowledge, only few online alternatives exist, which either employ older single-omic algorithms or are not accessible anymore. Although a variety of multi-omic and multi-view algorithms have been developed in recent years, they often lacked intuitive and comfortable handling. Web-rMKL makes the method more accessible for biomedical researchers and results could be used to design further biological experiments or in decision making of treatment in the case of cancer patients. The presented preprocessing tool further increases the accessibility of web-rMKL as it offers a way to easily compute kernel matrices with different kernel functions while keeping the sensitive data local.

In the future, we will integrate new model developments in the area, such as dealing with missing data, integrating localized multiple kernel learning as well as providing even more interpretable results.

## Supplementary Material

gkz422_Supplemental_Files

## References

[1] Speicher N.K., Pfeifer N. Integrating different data types by regularized unsupervised multiple kernel learning with application to cancer subtype discovery. Bioinformatics. 2015; 31:i268–i275. 26072491 10.1093/bioinformatics/btv244 PMC4765854

[2] Rappoport N., Shamir R. Multi-omic and multi-view clustering algorithms: review and cancer benchmark. Nucleic Acids Res. 2018; 46:10546–10562. 30295871 10.1093/nar/gky889 PMC6237755

[3] Witten D.M., Tibshirani R.J. Extensions of sparse canonical correlation analysis with applications to genomic data. Stat. Appl. Genet. Mol. Biol. 2009; 8:1–27. 10.2202/1544-6115.1470 PMC2861323 19572827

[4] Liu J., Wang C., Gao J., Han J. Multi-view clustering via joint nonnegative matrix factorization. Proceedings of the 2013 SIAM International Conference on Data Mining. 2013; SIAM 252–260.

[5] Mejía-Roa E., Carmona-Saez P., Nogales R., Vicente C., Vázquez M., Yang X., García C., Tirado F., Pascual-Montano A. bioNMF: a web-based tool for nonnegative matrix factorization in biology. Nucleic Acids Res. 2008; 36:W523–W528. 18515346 10.1093/nar/gkn335 PMC2447803

[6] Vert J.-P., Saigo H., Akutsu T. Convolution and local alignment kernels. Kernel methods in computational biology. 2004; 131–154.

[7] Yen-Yu Lin Y.-Y., Tyng-Luh Liu T.-L., Chiou-Shann Fuh C.-S. Multiple kernel learning for dimensionality reduction. IEEE Trans. Pattern Anal. Mach. Intell. 2011; 33:1147–1160. 20921580 10.1109/TPAMI.2010.183

[8] Yan S., Xu D., Zhang B., Zhang H.-j., Yang Q., Lin S. Graph embedding and extensions: a general framework for dimensionality reduction. IEEE Trans. Pattern Anal. Mach. Intell. 2007; 29:40–51. 17108382 10.1109/TPAMI.2007.12

[9] Gärtner T., Flach P.A., Kowalczyk A., Smola A.J. Multi-instance kernels. Proceedings of the Nineteenth International Conference on Machine Learning. 2002; San Francisco Morgan Kaufmann Publishers Inc 179–186.

[10] Goldman M., Craft B., Hastie M., Repečka K., Kamath A., McDade F., Rogers D., Brooks A., Zhu J., Haussler D. The UCSC Xena platform for public and private cancer genomics data visualization and interpretation. 2019; bioRxiv doi: 10.1101/326470, 05 March 2019, preprint: not peer reviewed.

[11] Daily K., Ho Sui S.J., Schriml L.M., Dexheimer P.J., Salomonis N., Schroll R., Bush S., Keddache M., Mayhew C., Lotia S. et al. Molecular, phenotypic, and sample-associated data to describe pluripotent stem cell lines and derivatives. Sci. Data. 2017; 4:170030. 28350385 10.1038/sdata.2017.30 PMC5369318

[12] Singh V.K., Kalsan M., Kumar N., Saini A., Chandra R. Induced pluripotent stem cells: applications in regenerative medicine, disease modeling, and drug discovery. Front. Cell Dev. Biol. 2015; 3:2. 25699255 10.3389/fcell.2015.00002 PMC4313779

