# Supplementary material for: web-rMKL: a web server for dimensionality reduction and sample clustering of multi-view data based on unsupervised multiple kernel learning
Source: Nucleic Acids Res. 2019 May 22;47(W1):W605–9. doi: 10.1093/nar/gkz422 (PMC6602472; doi:10.1093/nar/gkz422)
Supplement: gkz422_Supplemental_Files [file gkz422_supplemental_files.pdf]

web-rMKL: Supplementary File S1

Use case 1: TCGA cancer data

The TCGA data for head and neck squamous cell carcinoma (HNSC) was downloaded from the UCSC Xena browser (https://xenabrowser.net/datapages/?cohort=TCGA%20Head%20and%20Neck%20Cancer%20(HNSC), accessed at 2017-10-24). It comprised gene expression (IlluminaHiSeq), DNA methylation (Methylation450k), copy number variation (gistic2), and miRNA expression (IlluminaHiSeq) data, as well as clinical data.

For each data type, only solid tumor samples were retained. To avoid gender bias, we removed all features related to genes on the X and Y chromosome, if applicable. We then removed each feature with more than 20% missing values. Similarly, we removed each patient with more than 20% missing features. Remaining missing values were imputed using k nearest neighbor imputation considering k=3 nearest neighbors.

DNA methylation, measured using the Illumina Infinium HumanMethylation450 platform, was BMIQ normalized and aggregated into gene body and promoter methylation, each represented by the mean of the associated methylation sites.

Gene expression, copy number and miRNA expression data were standardized to a mean of zero and a variance of one.

Use case 2: Stem cell data

The normalized data for different cell lines were downloaded from https://www.synapse.org/pcbc (accessed at 2019-03-01). DNA methylation, gene expression and miRNA expression data were commonly available for 57 samples. If replicates were available, we combined the measurements by taking the median for each feature.
